# Heart rate variability as a strain parameter in maritime settings: a scoping review of methodological approaches to data collection and analysis in field studies on the high seas

**DOI:** 10.1186/s12995-026-00526-4

**Published:** 2026-08-21

**Authors:** Daniel Vasylenko, Lorenz Scheit, Chiara Reck, Dorothee Dengler, Volker Harth, Marcus Oldenburg

**Affiliations:** 1 Institute for Occupational and Maritime Medicine Hamburg (ZfAM), University Medical Center Hamburg-Eppendorf (UKE), 20459 Hamburg, Germany; 2 Bundeswehrkrankenhaus Hamburg, 22049 Hamburg, Germany

**Keywords:** Heart rate variability, HRV, Maritime medicine, Seafarers, Occupational health, Fatigue, Autonomic nervous system, Field study, Watchkeeping, LF/HF ratio

## Abstract

**Background:**

Seafarers are exposed to unique occupational stressors that place sustained demands on the autonomic nervous system. Heart rate variability (HRV) represents a promising non-invasive tool for assessing mental and physical strain in maritime settings. However, its methodological application in real field conditions remains poorly characterized, and existing HRV guidelines do not specifically address the methodological challenges of maritime field studies.

**Methods:**

A scoping review was conducted following PRISMA guidelines. Eleven maritime field studies measuring HRV in operational or semi-operational settings were included and assessed for device selection, measurement quality, analysis methods, HRV metrics, and covariates.

**Results:**

Chest strap systems were the most frequently used (*n* = 8) and showed sufficient validity for field use. Methodological heterogeneity in reporting of recording duration, analysis software, artifact handling and HRV parameters was high across studies. Operational factors including sailing schedule and work-rest organization were the most commonly examined covariates and were most frequently associated with significant HRV findings. Recurring findings were small and predominantly male populations, shared participant cohorts across multiple studies, and incomplete methodological reporting.

**Conclusion:**

Overall, the studies utilized HRV appropriately for their respective aims. HRV measurement in maritime field settings is feasible but methodologically demanding, and it remains an underexplored domain, which limits the conclusions that can be drawn. More detailed and standardized reporting is necessary. Based on the recurring methodological reporting gaps, this review proposes a maritime-specific reporting checklist and an overview of recommended methodological options for future studies. Larger study populations are needed to account for confounding factors on board ships.

**Supplementary Information:**

The online version contains supplementary material available at https://doi.org/10.1186/s12995-026-00526-4.

## Introduction

The maritime environment represents a uniquely demanding occupational setting. Seafarers are exposed to a wide range of physical and mental stressors that vary depending on their operational role on board, which can manifest in stress-related health impairments, cardiovascular strain, and reduced wellbeing [1, 2]. The work schedule on board, which often revolves around watchkeeping to ensure safe navigation, places sustained demands on the autonomic nervous system (ANS) and contributes to cumulative physiological and mental strain [3, 4].

Given that approximately 90% of global trade is transported by sea, the health and performance of seafarers carry substantial implications for maritime safety and global logistics [5]. In recent years, geopolitical instability has placed additional strain on international trade and maritime operations. The COVID-19 pandemic [6], international conflicts such as those in Ukraine or Iran [7], disruptions at maritime chokepoints like the Suez Canal [8] and maritime piracy [9] have further intensified stress on seafarers. The increasing workload of seafarers in this changing environment may impair cognition and raise the risk of accidents [8, 10, 11].

This raises the question of how this strain can be measured, quantified and analyzed in maritime conditions. A non-invasive method with high compliance and practical applicability is essential to obtain sufficient and valid data [12]. Heart rate variability (HRV) is a promising tool in this regard, allowing repeated or continuous measurement under real-world conditions through increasingly available portable devices [13]. When considering the future use of HRV as a strain parameter in maritime medicine, it is relevant to examine what prior experience exists with this measurement approach in shipboard studies. This includes their respective study design and the population investigated. This is essential not least to enable comparability across future maritime research.

The “Guideline for the application of heart rate and heart rate variability in occupational medicine and occupational health science” by Sammito et al. [14] links HRV measurement quality closely to both technical recording conditions and the chosen investigation methods. The technical implementation of HRV measurement at sea introduces further considerations. Device selection, particularly the distinction between ECG-based systems and chest-strap or wrist-based wearables, directly affects signal quality and applicability of these systems in the maritime environment [14]. The software used for HRV analysis, including the handling of artifacts and ectopic beats, similarly influences the comparability of results across studies [15]. With respect to quality criteria, Sammito et al. recommend: ECG recordings with a high sampling rate above 250 Hz [16], prior exclusion of relevant arrhythmia, and careful artifact inspection and correction. Recording length should be matched to the analytical method and study question, and in short-term recordings only a representative, quasi-steady, artifact-free segment should be used [14].

Applying these criteria in a maritime setting is challenging. Shipboard conditions (including vessel motion, vibrations, noise, limited space, fluctuating operational demands depending on the occupational role, and sleep deficits) may affect signal quality and must therefore be considered when planning HRV measurements at sea [2, 14]. These same shipboard conditions, however, are not only methodological constraints but also act as covariates. They continuously alter autonomic regulation, which is precisely what HRV captures. HRV is defined as the fluctuation in interbeat intervals (IBIs), also referred to as normal-to-normal (NN) intervals, between adjacent heartbeats, reflecting continuous modulation of the ANS [17, 18]. Time-domain, frequency-domain, and non-linear metrics are used to characterize HRV, with recording modes ranging from short-term recordings of approximately 5 min to long-term 24-hour assessments. Sammito et al. emphasized that both the chosen metric and the recording duration should always be matched to the respective study question [14, 19, 20]. This is in accordance with the guideline established by the Task Force of the European Society of Cardiology and the North American Society of Pacing and Electrophysiology as the framework for HRV usage in research [20]. Which of these metrics and recording modes have actually been applied in maritime field studies, and how they relate to the operational covariates of seafaring, therefore remains to be examined.

Against this background, this review aims to examine the use of HRV monitoring in maritime field studies, with particular attention to the applications and study designs employed, the measurement devices and analysis software used, adherence to established quality criteria, and the HRV metrics applied and their suitability compared with other parameters in the maritime context.

## Materials and methods

### PRISMA literature research

This scoping review examined the use of HRV measurement in maritime field studies. Conducted in accordance with the Joanna Briggs Institute (JBI) Manual for Evidence Synthesis [21], relevant literature was identified through a structured database search in PubMed (MEDLINE), Web of Science, CINAHL, and the Cochrane Library. The search strategy was developed in PubMed and adapted to the syntax requirements of the other databases. The search was structured around three groups of search terms: the maritime environment (e.g., seafarer, shipboard, naval, maritime, watchkeeping), HRV and related cardiac parameters (e.g., heart rate variability, HRV, RMSSD, SDNN, LF/HF, vagal tone, autonomic balance, ECG), and stress- or strain-related outcomes (e.g., stress, strain, fatigue, workload, job strain). The full search strings for all four databases are provided in Appendix A.

**Fig. 1:**
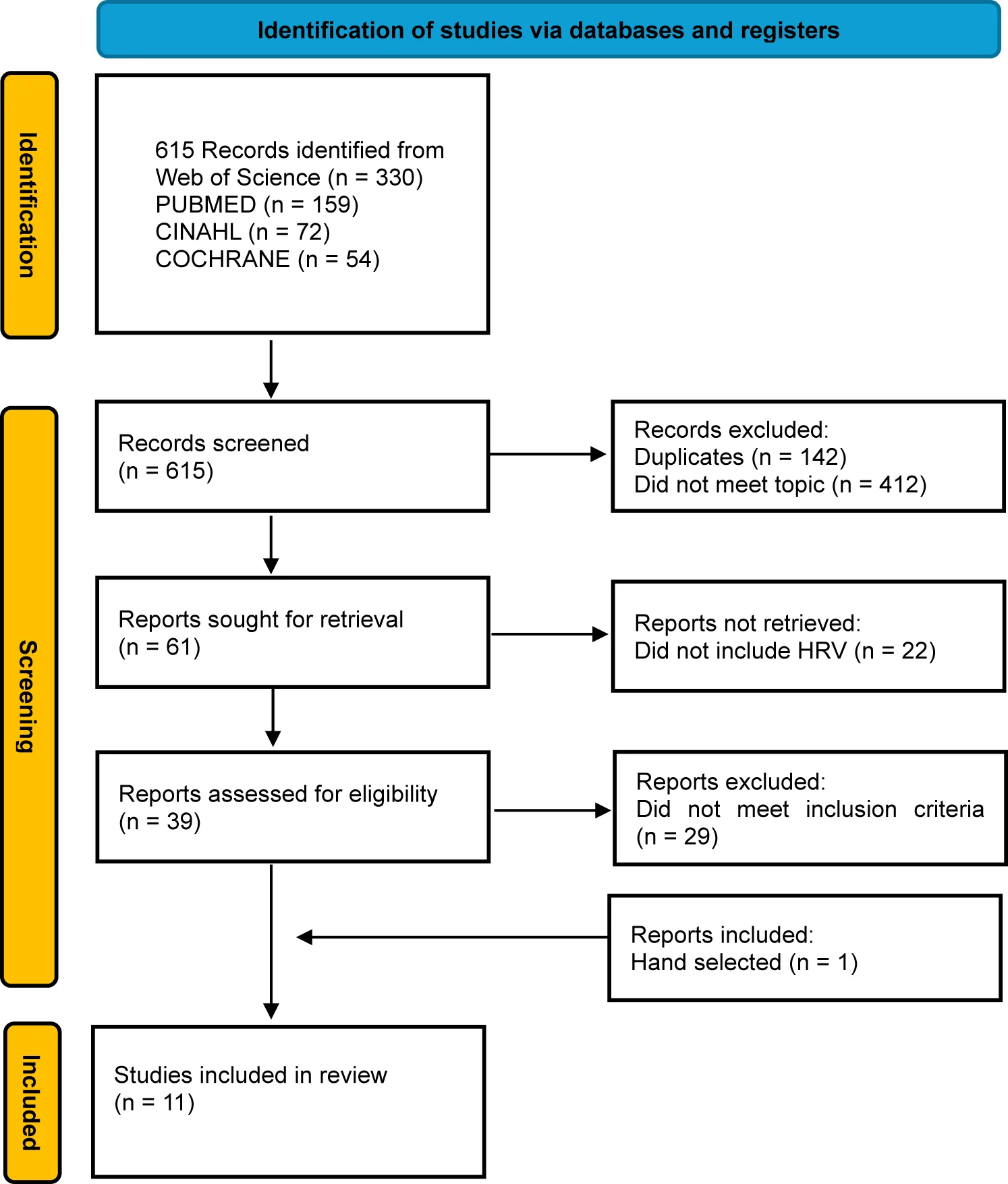
PRISMA flow chart

The final search identified 615 records, including 330 from Web of Science, 159 from PubMed (MEDLINE), 72 from CINAHL, and 54 from the Cochrane Library. Study selection was performed in accordance with the PRISMA Statement [22] (Fig. 1). During the initial screening process, 142 duplicate records were removed, and 412 records were excluded because they did not meet the topic of this review. Consequently, a total of 61 reports were sought for retrieval. Of these, 22 reports were not retrieved because they did not include HRV data, leaving 39 full-text reports to be assessed for eligibility. Studies were considered eligible if they investigated seafarers or seafaring personnel in maritime field studies and reported HRV data. A maritime field study was defined as a study in which data collection took place on board of a vessel, rather than in a laboratory or simulator environment. HRV measurement was defined as the assessment of beat-to-beat variation in cardiac intervals during the study period, reported either as standard time-domain, frequency-domain, or non-linear metrics, or as derived parameters based on these measures. Studies were excluded if they were not conducted in a real maritime field setting, were performed in a simulator environment, or did not report HRV data. After full-text assessment, 29 reports were excluded for not meeting the inclusion criteria. In total, eleven studies were included in the review, including one study that was identified manually.

### Data extraction and assessment criteria

The included studies were systematically assessed across five categories, each represented in a corresponding table. Table 1 summarized the general study characteristics, including study aim, study design, observation period, event profiling, and geographical setting, as well as methodological quality. Study design and risk of confounding were graded with the Scottish Intercollegiate Guidelines Network (SIGN) grading system. A three-level scale rating systematic reviews of case-control and cohort studies as well as case-control and cohort studies as 2++, 2+, or 2−, reflecting decreasing methodological quality and increasing risk of bias. Table 2 provided the population characteristics of the included studies, covering the type of population recorded, population size, age, sex distribution, rank or qualification level, working experience, nationality, and vessel type.

**Table 1:** Overview of characteristics of selected studies

Author (year)	Study Aim	SIGN Criteria	Study design	Observation period	Event profiling	Geography
Moraes et al. (2023) [23]	Assessing microbiome, inflammatory status and stress before, during and after an arctic summer camp	2+	Longitudinal study	62d total - 12d of ship travel and 50d of field camp	X	Antarctica, South America, Drake's passage
Oldenburg et al., 2019 (Voyage episodes) [24]	Comparing stress and strain amongst a sample of merchant seafarers across three voyage episodes	2-	Cross-sectional study	180 total investigation days biometric monitoring lasting an average of 2.8d (range: 2.5d to 6d)	X	Europe, North Sea, Baltic Sea
Yang et al. (2023) [25]	Investigating physiological, psychological and environmental factors on seafarers’ fatigue on passenger ships	2+	Longitudinal study	12d	X	Asia, China
Oldenburg et al. (2019) (Recreational) [26]	Investigating recreational possibilities during leisure time, effects on stress and strain, coping mechanisms among seafarers on a merchant ship	2-	Cross-sectional study	2.5d	X	Europe, international trade routes, North Sea
Moraes et al. (2020) [27]	Assessing hormonal, autonomic and mood state changes during an Antarctic expedition	2+	Longitudinal study	50d total − 26d of ship travel and 24d of field camp	X	Antarctica, South America
Özsever et al. (2018) [28]	Researching the human factor in maritime medicine by investigating which stress factors have the greatest impact on psychophysiological and cognitive aspects in seafarers on board of different vessels	2-	Cross-sectional study	1 cargo watch − 10-minute measurements taken exactly at the beginning and end of cargo watches	X	Turkey, Aegean
Kim et al. (2018) [29]	Assessing whether objectively measured cumulative stress and fatigue (via HRV) is correlated to subjective questionnaire-based surveys in seafarers	2+	Cross-sectional study	Single point measurement		Asia, South Korea
Oldenburg et al. (2019) (Occupational Groups) [30]	Analyzing stress and strain of seafarers between different occupational groups by surveying and biomonitoring	2-	Cross-sectional study	3d	X	Europe, North Sea, Baltic Sea
Ma et al. (2024) [31]	Developing a machine learning-based multimodal fusion recognition of seafarers’ workload with data gathered in a real navigation study	2+	Longitudinal study	24d	X	Asia, China
Myllylä et al. (2022) (Individual characteristics) [32]	Investigating individual characteristics of the naval personnel that may be associated with the amount of sleepiness, fatigue and stress responses experienced during shift work and irregular working hours	2+	Experimental crossover study	14d (2 × 7d)	X	Europe, North Sea
Myllylä et al. (2022) (Watchkeeping) [33]	Investigating the effect of a rotating 4:4 watch system to a 4:4/6:6 fixed watch system in terms of sleepiness, fatigue and stress responses	2+	Experimental crossover study	14d (2 × 7d)	X	Europe, North Sea

The technical aspects and measurement quality of HRV recordings were summarized in Tables 3 and 4. The measurement quality aspects in Table 4 were assessed via criteria extracted from the guideline of Sammito et al. Table 3 evaluated the monitoring mode of HRV, device category, device model, analysis software, sampling frequency, and evaluation interval. Signal and measurement quality was further assessed in Table 4 for participants’ pre-screening, resting baseline measurement, artifact and ectopic beat correction, circadian aspects, and an evaluation interval appropriate to the HRV parameters used. Pre-screening was rated as fulfilled when studies reported a medical pre-assessment intended to reduce relevant confounding (e.g., health certificates, medical examinations, or health questionnaires), and the remaining items were coded according to whether the respective procedure was reported. Table 5 captured the analytical scope of the included studies in terms of HRV metrics and covariates. HRV metrics were grouped into time-domain parameters (e.g., SDNN, Mean-RR, RMSSD), frequency-domain parameters (e.g., LF, HF, LF/HF), and study-specific or derived parameters. Covariates were categorized into operational factors (superscripts 1–5: work-rest schedule, sailing information/schedule, occupation/rank, ship type, workload assessment), environmental (superscript 6: sea state and weather), individual characteristics (superscripts 7–14: age, service time, fatigue and stress surveys, reaction time, behavior probability, physical fitness/activity, body biometrics, and personality/psychological assessment), and additional physiological measurements (superscripts 15–18: electroencephalography (EEG), sleep, electrodermal activity (EDA), and metabolic parameters).

### Declaration of the use of artificial intelligence

The use of artificial intelligence (AI) tools in the preparation of this manuscript complied with the relevant guidance of the University of Hamburg. ChatGPT 5.4^®^ was used for language support, specifically grammar correction, wording optimization, and improvement of text structure. NotebookLM^®^ was used for the organization of literature, overview of source content, and structuring of notes and extracted information. These tools served exclusively as supportive aids in the writing process and were not used to generate data or substitute for the author’s own scientific assessment. All interpretations, conclusions, and final formulations were made and verified by the author, who retains full responsibility for the manuscript.

## Results

### Study overview

The eleven studies selected for this review were maritime field studies that included HRV measurements in their assessment methods (Table 1). The study design of the selected studies comprised cross-sectional (*n* = 5), longitudinal (*n* = 4), and experimental crossover studies (*n* = 2). The observation period ranged from single-event recordings, such as cargo watches or shipboard examinations of seafarers, to multi-day recording periods lasting several days or weeks. All studies, besides the study of Kim et al., which involved a single point shipboard examination of seafarers, included event profiling in the form of a time frame, descriptions of events or references to the activities conducted during the investigation period (*n* = 10).

The categorization of the population is described in Table 2, where the population recorded comprised navy personnel, classic shipboard occupations, and expeditioners during Arctic expeditions. The studies included sample sizes ranging from 7 to 323 participants, with a median sample size of 24 across all studies, reflecting a predominance of small study populations. Age was provided in most studies (*n* = 10), whereas the reporting of sex was often limited by predominantly or exclusively male study populations. Occupational background was described either as rank-based categories such as officers and ratings or by operational roles, including deck officers, engine room personnel, technical staff, navy personnel, and expeditioners. Work experience was inconsistently reported (*n* = 4), and in naval studies was in some cases specified as prior naval service. Nationality or regional background was only documented in a subset of studies (*n* = 6) and ranged from single-national cohorts to more heterogeneous multinational crews. Overall, the reporting of demographic variables was heterogeneous and largely depended on the specific study aim and population under investigation. Across the included studies, research was conducted on a range of vessel types, comprising container or merchant ships (*n* = 5), navy polar ships (*n* = 2), passenger ships (*n* = 2), and military patrol boats (*n* = 2).

**Table 2:** Population characteristics of selected studies

Author (year)	Population recorded	Population size	Age	Sex	Rank / Qualification level	Working experience	Nationality	Type of ship
Moraes et al. (2023) [23]	Expeditioners	7	32.3 ± 8.4 yrs	5 male (71.4%), 2 female (28.6%)	Researchers, expeditioners	—	—	Navy polar ship
Oldenburg et al. (2019) (Voyage episodes) [24]	Seafarers (not specified)	323	38.2 ± 11.8 yrs	Exclusively male (100%)	—	—	—	Container ships
Yang et al. (2023) [25]	Deck / Nautical officers	24	37.52 ± 10.8 yrs	Exclusively male (100%)	Exclusively deck officers (100%)	13 ± 13.35 yrs	—	Passenger ships
Oldenburg et al. (2019) (Recreational) [26]	Deck / Nautical officers, deck ratings, engine room personnel, technical officers	323	37 yrs (range 18 - 71 yrs)	Exclusively male (100%)	37.8% (122) officers 62.2% (201) ratings	—	48.3% (156) Europeans 51.7% (167) Non-Europeans	Container ships
Moraes et al. (2020)[27]	Expeditioners	10	40 ± 11 yrs	7 male (70%), 3 female (30%)	Researchers, expeditioners	—	100% Brazilian	Navy polar ship
Özsever et al. (2018) [28]	Seafarers (not specified)	14	—	Exclusively male (100%)	—	—	—	Container ships
Kim et al. (2018) [29]	Deck / Nautical officers, deck ratings, engine room personnel, steward seamen, technical officers	125	59.62 ± 12.52 yrs	120 male (96%), 5 female (4%)	58.4% officers 36.8% ratings (seamen) 4.8% other positions	24.07 ± 13.28 yrs	100% (South-) Korean	Container ships, dangerous cargo ships, passenger ships, tugboats
Oldenburg et al. (2019) (Occupational Groups)[30]	Deck / Nautical officers, deck ratings, engine room personnel, technical officers	323	38.3 ± 11.8 yrs	Exclusively male (100%)	37.8% (122) officers 62.2% (201) ratings	—	48.0% Europeans 52.0% Southeast Asians	Container ships
Ma et al. (2024) [31]	Deck / Nautical officers	24	37.17 ± 10.48 yrs	Exclusively male (100%)	—	12.63 ± 12.91 yrs	—	Passenger ships
Myllylä et al. (2022) (Individual characteristics) [32]	Navy personnel	18	29 ± 8 yrs	Exclusively male (100%)	14 navy soldiers 4 conscripts	4.2 ± 5.4 yrs	100% Finns	Military patrol boat
Myllylä et al. (2022) (Watchkeeping) [33]	Navy personnel	15	29.1 yrs (range 19–45 yrs)	Exclusively male (100%)	12 navy soldiers 3 conscripts	—	100% Finns	Military patrol boat

### Technical recording methods and quality of recordings

The monitoring modes applied across the eleven included studies can be grouped into three main types of HRV assessment (Table 3). Four studies used scheduled time-point recordings, in which HRV was measured at predefined time points during the study period or as a single onboard assessment. Three studies applied multi-day continuous monitoring with 24-h recordings. Another three studies used event-bound monitoring, where HRV was recorded either during a specific event or before and after a defined task. Myllylä et al. reported a mixed design including both scheduled time-point recordings and multi-day 24-h recordings; however, only the scheduled time-point measurements were included in the reported results.

It should be noted that the three studies by Oldenburg et al. are based on the same underlying dataset but differ in their respective research focus. Similarly, the two studies by Myllylä et al. share a comparable study design but differ in their study population and research focus and may therefore be based on distinct measurement periods.

Polar^®^ devices (Polar Electro Oy, Kempele, Finland) were the most frequently used measurement systems, including the H10, S810i, V800, and RS800. Myllylä et al. used the Firstbeat^®^ Bodyguard 2 (Firstbeat Technologies Oy, Jyväskylä, Finland), representing the setup closest to a conventional ECG. Kim et al. used Omnifit^®^ Mindcare (Omni C&S, Seoul, Korea), a portable device combining EEG and photoplethysmography (PPG)-based pulse wave measurements to derive HRV-related indices. Kubios HRV^®^ (Kubios Ltd., Kuopio, Finland) was the most frequently reported analysis software, used in six studies, while Kim et al. relied on proprietary Omnifit^®^ software. Technical acquisition parameters were incompletely reported, with only four studies specifying a sampling rate ranging from 1000 to 5000 Hz. Evaluation intervals varied considerably, from 1-min short-term to 24-h long-term recordings.

With regard to signal and measurement quality, reporting was heterogeneous across studies (Table 4). Circadian rhythm was the most frequently addressed quality aspect, considered in nine studies, followed by pre-screening reported in eight studies. Recommended evaluation times were reported in seven studies, while resting baseline procedures and artifact correction were each described in six studies. A sufficient sampling rate was explicitly reported in only four studies. Regarding artifact correction specifically, threshold-based correction was the most commonly described approach. Özsever et al. additionally employed visual interval selection. Ma et al., Yang et al., Moraes et al., and Myllylä et al. all reported threshold-based artifact correction referencing a 3% threshold, without specifying a more precise correction level.

**Table 3:** Technical aspects and monitoring mode for HRV measurement in selected studies

Author (year)	Monitoring mode for HRV	Device category	Device model	Software used	Sampling rate (Hz)	Evaluation times
Moraes et al. (2023) [23]	Scheduled time-point recordings	Elastic electrode strap, wrist watch	Polar^®^ H10, Polar^®^ S810i	Kubios HRV^®^	Not reported	5-min
Oldenburg et al.(2019) (Voyage episodes) [24]	Multi-day continuous monitoring	wrist watch	Polar^®^ RS 800	Not mentioned	Not reported	24-h
Yang et al. (2023) [25]	Event-bound monitoring	Elastic electrode strap, wrist watch	Polar^®^ H10, Polar^®^ V800	Kubios HRV^®^ Version 3.5	5000 Hz	1-min data segments
Oldenburg et al. (2019) (Recreational) [26]	Multi-day continuous monitoring	Wrist watch	Polar^®^ RS 800	Not mentioned	Not reported	24-h
Moraes et al. (2020) [27]	Scheduled time-point recordings	Wrist watch	Polar^®^ S810i	Kubios HRV^®^	Not reported	5-min
Özsever et al. (2018) [28]	Event-bound monitoring	Elastic electrode strap	Polar^®^ chest belt (not specified)	Not mentioned	Not reported	10-min
Kim et al. (2018) [29]	Scheduled time-point recordings	Portable autonomic balance function analyzer	Omnifit^®^ Mindcare - Portable Type	On-board solution	Not reported	5-min
Oldenburg et al. (2019) (Occupational Groups) [30]	Multi-day continuous monitoring	Wrist watch	Polar^®^ RS 800	Not mentioned	Not reported	4-h & 24-h
Ma et al. (2024) [31]	Event-bound monitoring	Elastic electrode strap, wrist watch	Polar^®^ H10, Polar^®^ V800	Kubios HRV^®^ Version 3.4	5000 Hz	1-min
Myllylä et al. (2022) (Individual characteristics) [32]	Scheduled time-point recordings	Torso ECG electrodes	Firstbeat^®^ Bodyguard 2	Kubios HRV^®^ Version 3.4.3	1000 Hz	4-min
Myllylä et al. (2022) (Watchkeeping) [33]	Mixed	Torso ECG electrodes	Firstbeat^®^ Bodyguard 2	Kubios HRV^®^ Version 3.4.3	1000 Hz	4-min

**Table 4:** Measurement quality criteria for HRV analysis

Author (year)	Pre-Screening	Resting measurement	Artifact & ectopic beat Correction	Circadian rhythm addressed	Used recommended time for evaluation of HRV metrics chosen
Moraes et al. (2023) [23]	X	X	X	X	X
Oldenburg et al. (2019) (Voyage episodes) [24]				X	X
Yang et al. (2023) [25]	X	X	X		
Oldenburg et al. (2019) (Recreational) [26]				X	X
Moraes et al. (2020) [27]	X	X	X	X	X
Özsever et al. (2018) [28]	X			X	X
Kim et al. (2018) [29]	X			X	X
Oldenburg et al. (2019) (Occupational Groups) [30]				X	X
Ma et al. (2024) [31]	X	X	X		
Myllylä et al. (2022) (Individual characteristics) [32]	X	X	X	X	
Myllylä et al. (2022) (Watchkeeping) [33]	X	X	X	X	

Quality criteria were derived from the guideline of Sammito et al. Pre-screening was fulfilled when a medical pre-assessment was reported, for example by health certificates, medical examinations, or health questionnaires prior to participation. Recommended evaluation times were fulfilled when the analysis epoch matched the HRV metrics reported, in accordance with the guideline by Sammito et al

### HRV metrics and covariates associated with HRV

Regarding HRV metrics (Table 5), ten of the eleven studies reported classical time- and frequency-domain parameters. Among time-domain measures, the standard deviation of normal-to-normal intervals (SDNN) was most frequently applied (*n* = 7), followed by the root mean square of successive differences (RMSSD) (*n* = 5), mean RR interval (Mean-RR) (*n* = 3), and the number/percentage of successive NN interval differences greater than 50 ms (NN50/pNN50) (*n* = 2). In the frequency domain, the low-frequency to high-frequency ratio (LF/HF) was the most frequently reported parameter (*n* = 7), followed by high-frequency power (HF) (*n* = 5), low-frequency power (LF) (*n* = 4), total power (TP) (*n* = 2), and very low-frequency power (VLF) (*n* = 1). Two studies reported derived parameters: Moraes et al. used the logarithm of RMSSD, while Kim et al. reported a proprietary device-derived cumulative fatigue index associated with autonomic function and reduced HRV.

Concerning covariates, sailing information or schedule (superscript 2 in Table 5) was the most frequently assessed covariate (*n* = 5), referring to voyage-related characteristics such as departure times, number of port visits, or the overall period of sea travel. Work-rest schedule¹ was assessed in four studies, encompassing watchkeeping systems, differences between working and resting periods, and circadian rhythm-related aspects. Occupation or rank³ was the third most frequently examined covariate (*n* = 3), mainly comparing HRV between different professional or hierarchical groups on board. Fatigue and stress surveys⁹, age⁷, EEG¹⁵, and EDA¹⁷ were each assessed in two studies (*n* = 2), while the remaining covariates were reported only once. Overall, the assessment of covariates alongside HRV was highly heterogeneous, both regarding the parameters considered and the HRV metrics used for analysis.

Significant associations were most frequently observed for SDNN, Mean-RR, RMSSD, HF, and LF/HF, primarily in relation to operational covariates such as sailing schedule² and work–rest organization¹, but also for age⁷, fatigue and stress surveys⁹, EEG¹⁵, EDA¹⁷, and individual characteristics. In Yang et al. (2023), Mean-RR and LF/HF emerged as significant HRV predictors in ordinal logistic regression. In Özsever et al. (2018), frequency of circadian rhythm change was retained as the significant determinant of LF/HF in multiple regression. Ma et al. (2024) analyzed HRV features within a multimodal machine-learning model, where LF/HF and Mean-RR ranked among the most relevant predictors. Myllylä et al. (2022) reported significant associations across a wider range of HRV metrics in relation to watchkeeping schedule¹ and individual characteristics. In summary, operational covariates provided the most frequent context for significant HRV findings, while regression and machine-learning approaches consistently reduced these to a smaller subset of key predictors.

**Table 5:** HRV metrics assessed in relation to covariates across the included maritime field studies

	Time domain					Frequency domain				
	SDNN (*n* = 7)	Mean RR (*n* = 3)	RMSSD (*n* = 5)	NN50 (*n* = 2)	pNN50 (*n* = 2)	LF (*n* = 4)	HF (*n* = 5)	LF/HF (*n* = 7)	Less frequently reported	
Moraes et al. (2023) [23]	X^2^	**X** ^**2**^	X^2^	X^2^	X^2^	X^2^	X^2^	X^2^	Ln (RMSSD)^2^	
Oldenburg et al. (2019) (Voyage episodes) [24]	X^2^									
Yang et al. (2023) [25]	**X** ^**2**,6,7,**9**,15^	**X** ^**2**,**6**,**7**,**9**,**15**^	**X** ^**2**,6,**7**,**9**,15^				**X** ^**2**,**6**,7,**9**,15^	**X** ^**2**,6,**7**,**9**,**15**^		
Oldenburg et al. (2019) (Recreational) [26]	X^1,3,16^									
Moraes et al. (2020) [27]			**X** ^**2**^	X^2^	X^2^	X^2^	**X** ^**2**^	**X** ^**2**^		
Özsever et al. (2018) [28]								**X** ^1,2,**10**,**17**^		
Kim et al. (2018) [29]									Cumulative fatigue index^3,4,9^	
Oldenburg et al. (2019) (Occupational Groups) [30]	**X** ^**3**^									
Ma et al. (2024) [31]		**X** ^**15**^						**X** ^**5**,**11**,**15**,**17**^		
Myllylä et al. (2022) (Individual characteristics) [32]	**X** ^**1**,**8**,**12**,**14**,**18**^		**X** ^**1**,**12**,**14**,**18**^			**X** ^**7**,**12**,**13**,**14**,**18**^	**X** ^**1**,**18**^	**X** ^**1**,**8**,**12**,**14**^	**TP** ^**1**,**8**,**12**,**13**,**18**^	
Myllylä et al. (2022) (Watchkeeping) [33]	X^1^		X^1^			**X** ^**1**^	X^1^	X^1^	VLF^1^, TP^1^	

HRV metrics are grouped into time-domain parameters (SDNN, Mean-RR, RMSSD, NN50, pNN50) and frequency-domain parameters (LF, HF, LF/HF), with additional study-specific indices listed under “Less frequently reported”. Superscript numbers indicate the covariates with which the respective HRV metric was assessed, grouped by category - **operational factors**: ¹work-rest schedule, ²sailing information/schedule, ³occupation/rank, ⁴ship type, ⁵workload assessment; environmental factors: ⁶sea state and weather; **individual characteristics**: ⁷age, ⁸service time, ⁹fatigue and stress surveys, ¹⁰reaction time, ¹¹behavior probability, ¹²physical fitness/activity, ¹³body biometrics, ¹⁴personality/psychological assessment; **additional physiological measures**: ¹⁵EEG, ¹⁶sleep (HRV measurement during sleep or compared with sleepiness), ¹⁷EDA, and ¹⁸metabolic parameters

**Bold** entries and **underlined superscript** indicate statistically significant associations between the respective HRV metric and covariate (*p* ≤ 0.05)

Abbreviations: HRV, heart rate variability; SDNN, standard deviation of normal-to-normal intervals; Mean-RR, mean RR interval; RMSSD, root mean square of successive differences; LF, low-frequency power; HF, high-frequency power; LF/HF, ratio of low-frequency to high-frequency power; NN50, number of successive normal-to-normal interval differences greater than 50 ms; Ln(RMSSD), natural logarithm of RMSSD; TP, total power; VLF, very low frequency; EEG, electroencephalography; EDA, electrodermal activity

## Discussion

### Technical measurement methods

#### Device selection and sampling rate (Hz)

Most studies in this review used Polar^®^ products to record HRV (Table 3). The Polar S810i, RS800, and V800 all share the same functional principle: a chest belt detects cardiac electrical activity and wirelessly transmits beat-to-beat intervals to a wrist-worn receiver unit. None of these devices use optical pulse measurement at the wrist, meaning the recorded signal originates from cardiac conduction rather than peripheral blood flow [34–36]. This is relevant, as wrist-worn devices that measure the pulse assess pulse rate variability (PRV) rather than the actual HRV. A systematic review showed that PRV is only reliably comparable to HRV in young, healthy subjects under resting conditions [37]. Given their widespread use and continuous data output, wearables like smartwatches and fitness trackers warrant further investigation in the maritime field [38].

The most used device in the studies included was the Polar^®^ H10 chest strap, which has shown good reliability (intraclass correlation coefficient (ICC)₃,₁ = 0.95 at rest and ICC₃,₁ > 0.93 during incremental exercise) in measurement of HRV in comparison with a 12-lead ECG during rest and exercise. Furthermore, it proved suitable for field studies involving varying levels of physical activity [39]. In addition, the Polar^®^ systems mentioned above were shown to have good reliability compared with traditional ECGs, with ICC values of 0.869–0.995 across HRV parameters at rest and during exercise [40–42]. Oldenburg et al. [24] similarly observed high validity for the RS800 worn by four examiners over three days on board and ashore.

The only ECG-based system used was the Firstbeat^®^ Bodyguard 2, applied with adhesive electrodes comparable to ECG lead II and showing good validity. This is in line with current guideline recommendations that ECG-based systems should be preferred over chest strap systems where applicable [14, 43].

Sampling frequency is an important factor in device selection, with a minimum of 250 Hz recommended for HRV measurement [14, 16, 20]. For example, the Polar^®^ H10 device manual states that it is capable of a frequency of 5000 Hz [44], this is also stated in the studies of Ma et al. and Yang et al. [25, 31]. The Firstbeat^®^ Bodyguard 2 device exceeds 250 Hz with a sampling rate of 1000 Hz according to the manufacturer specifications [45].

The devices used generally fulfill the requirements for measuring HRV in field studies. However, the limitations of chest strap devices must be considered in future studies. They do not provide a full ECG recording and record NN intervals with varying accuracy, which can cause deviations in subsequent HRV analysis [14]. Where feasible, ECG-based systems should be preferred; validated chest-strap devices are the most practical alternative for maritime field studies, whereas optical wrist devices are not interchangeable with ECG-derived HRV.

#### Quality aspects in measurement

HRV measurement in maritime medicine is influenced by shipboard conditions: vibrations, ship motion, noise, limited space and fluctuating operational demands [2, 14]. These factors should therefore be considered as methodological limitations and potential confounders, as illustrated by the included studies. Moraes et al. reported a reduced final sample size during ship travel due to “limited and low-quality signal acquisition” that led to “excessive interference or significant capture gaps, resulting in less than 5 min of good quality signal” [27]. Oldenburg et al. [26] observed a lower HRV during sleeping hours and suggested that “permanent heaving motions of the vessel, noise and vibration” were possible confounders. Myllylä et al. stated that it is “impossible to achieve laboratory-like conditions” on board due to varying weather and vessel operations [33].

This shows that not only device choice matters, but also how clearly methodological procedures were described and performed to reduce confounders. Methodological reporting was highly heterogeneous: resting conditions, electrode fitting, participant pre-screening and recording environment varied considerably or were inadequately described [14]. Good examples were the studies of Özsever et al., Yang et al. and Ma et al. [25, 28, 31], who provided pictures of application of the device and how the measurement under operational conditions looked. Demographic and occupational differences between study populations should also be considered, as HRV is influenced by age and sex [46].

#### Kubios^®^ utilization and artifact clearance

An overview of Tables 3 and 5 shows that studies using Kubios^®^ reported a wider range of HRV metrics. Additionally, more of these metrics reached statistical significance compared with studies that did not report Kubios^®^. This does not imply that Kubios^®^ is the most viable HRV software, but rather that dedicated HRV software may improve analysis variety and quality through its metric range and artifact handling. For this reason, the software used for HRV analysis must always be reported, ideally together with the software version, as updates may alter preprocessing steps or analytical procedures [47].

The same applies to artifact correction, with threshold-based methods being the most commonly reported approach, consistent with the standard function of Kubios^®^. This method works by comparing a NN interval to a local average and if the NN interval exceeds the set threshold compared with the local average it is excluded. Notably, Kubios^®^ offers multiple predefined correction levels ranging from “very low” to “very strong,” as well as a custom option [48]. Previous studies have shown that overcorrection can lead to low HRV results, while undercorrection may lead to a high HRV, affecting the HRV analysis [15, 49]. None of the studies reporting threshold-based artifact correction specified the exact threshold level applied, which should be considered a reporting gap given its potential influence on HRV outcomes.

Given the shipboard conditions described above, artifact handling is of particular importance in maritime HRV field studies. Beyond threshold correction, Kubios^®^ Premium offers noise detection with automatic exclusion of noisy segments [48]. The guideline by Sammito et al. recommends the handling of artifacts via visual inspection, manual correction or the stated data processing whenever possible [14]. It can be recommended that dedicated HRV software should be used in future studies and the handling of their features, such as artifact handling should be reported accordingly.

### Metrics and covariates

#### HRV metrics and recording modes

The selection of HRV metrics was appropriate to the research objectives and consistent with the recording durations used, as recommended by Sammito et al. and the European Task Force [14, 20].

SDNN was the most frequently applied time-domain metric (*n* = 7), reflecting sympathetic and parasympathetic influences and applicable to short- and long-term recordings [14, 19, 20]. In the Oldenburg studies [24, 26, 30], 24-hour monitoring used SDNN to assess autonomic tone across voyage episodes and occupational groups, whereas the shorter recordings by Moraes et al. [23, 27] and Myllylä et al. [32, 33] applied it in relation to sailing schedules and individual characteristics. RMSSD (*n* = 5) is a parasympathetic mediated parameter suited for short-term measurements [14, 19]. Its sensitivity suits analyses of autonomic recovery across sailing episodes and work-rest cycles, as in Moraes et al. [27] and Myllylä et al. [32, 33].

In the frequency domain, LF/HF ratio (*n* = 7) was most frequently used. It is the quotient of low- and high-frequency spectral power and has no clear alignment with ANS activity [14, 19]. LF/HF ratio, while widely applied, remains a controversial indicator of sympathovagal balance and should be interpreted with caution [50]. LF and HF are shaped by multiple context-dependent influences and do not represent direct, reciprocal measures of sympathetic and parasympathetic activity; their ratio therefore describes relative autonomic modulation rather than actual balance [20, 50, 51]. Its predictive weight reflects sensitivity to the conditions under investigation rather than validated physiological meaning [50, 51, 52]. Modern HRV software such as Kubios^®^ increasingly uses non-linear parameters like Poincaré plot derived SD1 and SD2 as indicators of parasympathetic and sympathetic tone, which are currently not represented in the included studies [48, 53]. HF (*n* = 5) reflects parasympathetic activity via respiratory sinus arrhythmia, whereas LF (*n* = 4) is influenced by both sympathetic and parasympathetic activity, although this assignment remains debated [14, 20, 54]. Frequency-domain parameters require a minimum recording duration of five minutes for reliable spectral analysis [14, 20]. Özsever et al. [28] and Moraes et al.[23, 27] met the five-minute minimum, whereas the one-minute epochs of Ma et al. and Yang et al. [25, 31] and the four-minute epochs of Myllylä et al. [32, 33], fell below this threshold, which limits the interpretability of their frequency-domain results. No single recording duration is universally preferable for maritime HRV research; methodological quality depends on whether the interval fits the research question and the HRV parameters interpreted [14].

#### Covariates

The covariates most frequently examined alongside HRV reflect seafarers occupational reality: operational factors such as sailing schedule, work-rest organization, and occupation dominate, as these are the primary sources of physiological strain at sea [2]. Where assessed, individual characteristics (including age, physical fitness, metabolic parameters, and personality) have been shown to be relevant across a broad range of HRV metrics [32]. This underlines that autonomic regulation at sea is shaped by both work conditions and individual characteristics. These factors should be systematically considered in future studies, in line with the Sammito et al. guideline [14].

Moraes et al. observed a biphasic HRV pattern across voyage episodes. RMSSD declined by 47.8% before recovering toward journey’s end. The LF/HF ratio followed a similar pattern alongside elevated urinary catecholamines, though without statistical testing [27]. This suggests episode-specific conditions and stress hormones are relevant covariates for future HRV research.

Three studies applied multivariate approaches to identify the most relevant HRV predictors. In Yang et al.‘s ordinal logistic regression, LF/HF and mean-RR were the strongest predictors of fatigue (81.1% accuracy) [25]. Özsever et al. applied multiple regression to link operational factors and drowsiness. LF/HF and EDA correlated with circadian rhythm changes, indicating that HRV can capture autonomic responses to watchkeeping-induced schedule disruption [28]. Ma et al. integrated HRV into a machine-learning model, reaching 85.7% accuracy in predicting fatigue. LF/HF ranked as the highest-weighted HRV feature [31]. All three approaches demonstrate that HRV parameters can serve as reliable features for predictive systems. HRV integration into AI-based biomonitoring holds promise for real-time fatigue detection, incident prevention, and human performance optimization at sea [55, 56].

### Critical appraisal and recommendations for standardization

Based on the findings of this review, two major methodological concerns currently stand out for the maritime field:

The first concern is selection bias, which is barely avoidable in maritime field studies. Recruitment depends on who is on board, so crew size and demographic composition limit comparability between studies. Population sizes ranged from 7 to 323 with a median of 24, showing a predominance of small samples. Most studies also recorded exclusively or predominantly male crews. While this reflects a still male-dominated profession, female seafarers remain underexplored [57]. Findings from male-only samples cannot be generalized to women, whose cardiac autonomic profile differs [46, 58].

The second, affecting every included study to some degree, is incomplete reporting (Tables 3 and 4). A procedure that is not described cannot be distinguished from one that was not performed, which also makes further sources of bias, such as measurement bias or unreported confounding, harder to exclude [51, 59].

Despite these limitations, the included studies advance HRV research in field studies and provide an initial empirical foundation for this emerging field. A structured overview of recommended methodological options to implement HRV in field studies is provided in the supplementary material.

Structured HRV reporting frameworks exist for adjacent fields [60, 61], but do not address maritime measurement. More independent studies and more consistent reporting are needed to strengthen the evidence base. To close these gaps, Table 6 additionally provides a maritime-specific reporting set comprising six domains that follow the order of this review: study design and population (A), device and measurement (B), recording duration and monitoring mode (C), data processing and artifact correction (D), HRV metric selection (E), and covariates and confounders (F). The recommended reporting items for each domain are specified in Table 6 [14].

**Table 6:** Checklist of recommended reporting items when implementing HRV in maritime field studies

	Item	Recommended reporting
**A**	**Study design and population**	
A1	Maritime specific reporting of study design	**Vessel type**, **voyage route**, if applicable kind of operations conducted
A2	Population and occupational role	General: **size**, **age**, **sex**, nationality, biometric data Maritime-specific: **department**, **rank**, occupational experience as seafarers
**B**	**Device and measurement**	
B1	Device category	**State the type of system used**,** for example chest strap system**,** ambulatory torso ECG**,** stationary ECG**,** wrist watch or other wearable**
B2	Device model	**Manufacturer and model**, if applicable firmware version
B3	Sampling rate	**Sampling frequency in Hz of the recorded data**
B4	Device Handling	**Describe how the device was used on board**. Document the handling steps relevant to quality control, such as skin preparation, electrode placement and device adjustment
B5	Device compliance	**Number of recordings started versus those available for analysis**, with reasons for loss (for example device removed during work, strap detachment, data transfer failure)
**C**	**Recording duration and monitoring mode**	
C1	Monitoring mode	**Short-term or long-term recording** **Single time point measurement**,** repeated measurements**,** pre-post measurements or single to multiday recordings**
C2	Recording length	**State recording length or length of investigated HRV segment**
C3	Recording procedure	Step-by-step description of how the measurement was carried out on board, and who applied the device and with what training Photographic or schematic documentation of the measurement setup where permitted by the operator
C4	Time of day and circadian control	**State the time of day at which the measurement was conducted and how circadian variation was accounted for**
**D**	**Data processing and artifact correction**	
D1	Analysis software	**State software name**,** version number**,** developer**
D2	Artifact correction	**State the artifact correction method and the correction level or threshold applied**
D3	Data quality	Where low-quality data occurred, document how it was handled and which sources of disturbance were identified. State the number of corrected beats or the proportion of excluded segments where the software provides this information
D4	Further software-specific reporting	Report whether proprietary software features, indices or algorithms were used
**E**	**HRV metrics selection**	
E1	Metrics and indices	**Full list of the metrics used**, preferably in absolute units rather than as relative change only. Also report other forms of HRV description, such as proprietary indices
E2	Frequency domain calculation	**Report how frequency-domain metrics were calculated** (spectral estimation method, for example fast-Fourier transformation or autoregressive modelling, and the frequency band limits applied)
**F**	**(Maritime) covariates and confounders**	
F1	Operational factors	Work and rest schedule, watch system, voyage or sailing schedule, port versus sea phase, rank or occupation, ship type, workload assessment
F2	Environmental factors	Sea state, weather, vibration, noise, ambient temperature and workplace conditions
F3	Individual factors	Age, sea service time, physical activity and fitness, body biometrics, sleep, fatigue and stress questionnaires
F4	Acute confounders	Medication, caffeine, nicotine, alcohol, meals, seasickness and antiemetic use, acute illness, physical exertion shortly before the recording
F5	Health pre-screening	How medical fitness was documented (seafarer medical certificate, examination, questionnaire) and which cardiac conditions or medications led to exclusion

**Bold** entries are categorized as the minimal mandatory reporting items according to the findings of the present review. Reporting items in the domain *F Covariates and Confounders* can not be sorted into mandatory categories as those items can be highly specific to study design, whilst all can act as confounders

### Limitations

Most included studies used cross-sectional designs with relatively small sample sizes, which limits generalizability. The studies differed considerably in recording duration, devices, and reported parameters, making direct comparisons challenging. Some studies are based on the same cohorts, meaning these populations receive greater weight in the overall findings. Where information was not mentioned in the studies, it cannot necessarily be concluded that these aspects were neglected (such as device handling). The broader field remains dominated by simulator-based research, and field studies in real operational environments are still scarce.

Furthermore, the study of Kim et al. was published in Korean, and assessment relied on a version translated with an AI-assisted tool, which may have introduced inaccuracies in the interpretation of methodological details and results.

HRV interpretation is still subject to ongoing scientific discussion, and no universally accepted standards for maritime medicine can be derived at this point. Seafarer stress is inherently multifactorial, which makes it difficult to establish clear causal links to HRV findings. In particular, which HRV metrics are most suitable for which covariate in maritime medicine remains an open question, as the limited number of available studies does not yet allow evidence-based recommendations for metric selection tailored to specific covariates. Additionally, physical interference from the shipboard environment (such as vessel motion or engine vibration) cannot be excluded as a potential source of signal distortion.

## Conclusion

This review shows that HRV measurement under real maritime conditions is feasible but methodologically demanding.

The included studies suggest that operational and occupational factors represent the primary sources of autonomic strain at sea, reflected in consistent HRV changes across studies. However, maritime field research remains underexamined, and the heterogeneity in device selection, software, artifact correction, metrics, and covariates assessed limits comparability and prevents firm conclusions about the relationship between these factors and HRV under real operational conditions.

Methodological standardization is therefore the most pressing need for this field. Future studies should align device selection, recording duration, artifact correction, and HRV parameter reporting with established guidelines, ensuring that the chosen metrics are appropriate to the research question and recording duration. To support this, a reporting checklist and an overview of recommended methodological options for field studies are proposed in this review to summarize the main methodological and reporting recommendations for future studies. Larger and more diverse study populations are needed to improve generalizability. The integration of HRV into AI-based monitoring systems remains a promising long-term direction for fatigue and stress detection in maritime settings, but further methodologically sound field research is required before reliable implementation becomes possible.

## Appendix

### Appendix A – Search strings

#### PUBMED

( “Ships“[Mesh] OR “Naval Medicine“[Mesh] OR “shipboard“[Title/Abstract] OR “on board“[Title/Abstract] OR “onboard“[Title/Abstract] OR “seafarer*“[Title/Abstract] OR “sailor*“[Title/Abstract] OR “seamen“[Title/Abstract] OR “seaman“[Title/Abstract] OR “ship crew“[Title/Abstract] OR “merchant ship“[Title/Abstract] OR “merchant vessel“[Title/Abstract] OR “container ship“[Title/Abstract] OR “fishing vessel“[Title/Abstract] OR “nautical officer*“[Title/Abstract] OR “maritime“[Title/Abstract] OR “at sea“[Title/Abstract] OR “watchkeep*“[Title/Abstract] OR ((Navy[Title/Abstract] OR naval[Title/Abstract]) AND (ship*[Title/Abstract] OR vessel*[Title/Abstract] OR boat*[Title/Abstract] OR submarine*[Title/Abstract] OR “at sea“[Title/Abstract] OR onboard[Title/Abstract])) OR (pilot*[Title/Abstract] AND (maritime[Title/Abstract] OR harbor[Title/Abstract] OR marine[Title/Abstract])) ) AND ( “Heart Rate Variability“[Mesh] OR “Heart Rate“[Mesh] OR “HRV“[Title/Abstract] OR “heart rate variability“[Title/Abstract] OR “PRV“[Title/Abstract] OR “pulse rate variability“[Title/Abstract] OR “RMSSD“[Title/Abstract] OR “SDNN“[Title/Abstract] OR “RR interval“[Title/Abstract] OR “R-R interval“[Title/Abstract] OR “LF/HF“[Title/Abstract] OR “vagal tone“[Title/Abstract] OR “autonomic balance“[Title/Abstract] OR “cardiac autonomic“[Title/Abstract] OR “heart rate“[Title/Abstract] OR “HR“[Title/Abstract] OR “ECG“[Title/Abstract] OR “electrocardio*“[Title/Abstract] OR “CPL“[Title/Abstract] OR “cardiac strain“[Title/Abstract] ) AND ( “stress“[Title/Abstract] OR “strain“[Title/Abstract] OR “fatigue“[Title/Abstract] OR “workload“[Title/Abstract] OR “mental workload“[Title/Abstract] OR “work stress“[Title/Abstract] OR “job strain“[Title/Abstract] )

#### Web of Science

(TS=(shipboard OR “on board” OR onboard OR seafarer* OR sailor* OR seamen OR seaman OR “ship crew” OR “merchant ship” OR “merchant vessel” OR “container ship” OR “fishing vessel” OR “nautical officer*” OR maritime OR “at sea” OR watchkeep* OR watchkeeping OR Ships OR “Naval Medicine”) OR (TS=(Navy OR naval) AND TS=(ship* OR vessel* OR boat* OR submarine* OR “at sea” OR onboard)) OR (TS=(pilot*) AND TS=(maritime OR harbor OR marine)) ) AND TS=(“Heart Rate Variability” OR HRV OR “pulse rate variability” OR PRV OR RMSSD OR SDNN OR “RR interval” OR “R-R interval” OR “LF/HF” OR “vagal tone” OR “autonomic balance” OR “cardiac autonomic” OR “Heart Rate” OR HR OR ECG OR electrocardio* OR CPL OR “cardiac strain”) AND TS=(stress OR strain OR fatigue OR workload OR “mental workload” OR “work stress” OR “job strain” OR recovery).

#### COCHRANE

**Table:** 

ROW	Cochrane Syntax
**#1**	(sailor* OR seafarer* OR seamen OR seaman OR naval OR ship OR shipping OR ships OR maritime): ti, ab
**#2**	(“Heart Rate Variability” OR HRV OR “pulse rate variability” OR PRV OR RMSSD OR SDNN OR “RR interval” OR “R-R interval” OR “LF/HF” OR “vagal tone” OR “autonomic balance” OR “cardiac autonomic” OR “Heart Rate” OR HR OR ECG OR electrocardio* OR CPL OR “cardiac strain”):ti, ab
**#3**	**#1 AND #2**

#### CINAHL

(TI (sailor* OR seafarer* OR seamen OR seaman OR naval OR ship OR shipping OR ships OR maritime) OR AB (sailor* OR seafarer* OR seamen OR seaman OR naval OR ship OR shipping OR ships OR maritime)) AND (TI (“Heart Rate Variability” OR HRV OR “pulse rate variability” OR PRV OR RMSSD OR SDNN OR “RR interval” OR “R-R interval” OR “LF/HF” OR “vagal tone” OR “autonomic balance” OR “cardiac autonomic” OR “Heart Rate” OR HR OR ECG OR electrocardio* OR CPL OR “cardiac strain”) OR AB (“Heart Rate Variability” OR HRV OR “pulse rate variability” OR PRV OR RMSSD OR SDNN OR “RR interval” OR “R-R interval” OR “LF/HF” OR “vagal tone” OR “autonomic balance” OR “cardiac autonomic” OR “Heart Rate” OR HR OR ECG OR electrocardio* OR CPL OR “cardiac strain”)).

## Supplementary Information

Below is the link to the electronic supplementary material.


Supplementary Material 1


## Data Availability

All data generated or analyzed during this study are included in the manuscript and its supplementary information files. The full search strings used for the database search are provided in Appendix A.

## Abbreviations

AI: Artificial intelligence
ANS: Autonomic nervous system
BMI: Body mass index
CINAHL: Cumulative Index to Nursing and Allied Health Literature
ECG: Electrocardiogram
EDA: Electrodermal activity
EEG: Electroencephalography
HF: High-frequency power
HRV: Heart rate variability
IBI: Interbeat interval
ICC: Intraclass correlation coefficient
JBI: Joanna Briggs Institute
LF: Low-frequency power
LF/HF: Ratio of low-frequency to high-frequency power
Mean-RR: Mean RR interval
MEDLINE: Medical Literature Analysis and Retrieval System Online
NN: Normal-to-normal (intervals)
NN50: Number of successive NN interval differences greater than 50 ms
pNN50: Percentage of successive NN interval differences greater than 50 ms
PPG: Photoplethysmography
PRISMA: Preferred Reporting Items for Systematic Reviews and Meta-Analyses
PRV: Pulse rate variability
RMSSD: Root mean square of successive differences
SD1/SD2: Poincaré plot derived short- and long-term variability indices
SDNN: Standard deviation of normal-to-normal intervals
SIGN: Scottish Intercollegiate Guidelines Network
TP: Total power
VLF: Very low-frequency power
