# Supplementary material for: Heart rate variability as a strain parameter in maritime settings: a scoping review of methodological approaches to data collection and analysis in field studies on the high seas
Source: J Occup Med Toxicol. 2026 Aug 21;21:26. doi: 10.1186/s12995-026-00526-4 (PMC13528020; doi:10.1186/s12995-026-00526-4)
Supplement: Supplementary file 1 [file 12995_2026_526_MOESM1_ESM.docx]

## Structured overview of recommended methodological options for HRV measurements in field studies

| **1 Measurement mode & study concept** – driven by the research question and by operational reality on board | |
| --- | --- |
| **Long-term (24 h)** **Use:** overall variability (not event-bound), daily HRV monitoring, 24-h environmental impact **Strengths:** single artifacts don’t have as big of an impact due to long recording; continuous recording, no constant researcher attention needed; covers circadian rhythm when sleep cycle included **Limits:** specific covariates and conditions are more difficult to record; longer recordings; compliance can drift over 24 h | **Short-term (≥5 min)** **Use:** pre/post assessment of occupational procedures  Cross-sectional; Repeated / Longitudinal  **Strengths:** controlled conditions (same time, place, population); scales impact of defined events; more precise devices available **Limits:** availability varies with occupational duties; more vulnerable to motion, noise, vibration, EMI; often needs research personnel |

↓

| **2 Device choice** – sampling rate ≥250 Hz validated at rest *and* exercise / low-interference and self-applicable if no researcher is present / must fit constraints of workspace | | |
| --- | --- | --- |
| **Device** | **Profile** | **Recommendation** |
| **Portable 24-h ECG** | Real ECG, low interference; best mix of daily applicability and data quality; usually needs researchers for lead placement | **Recommended if available** |
| **Stationary 12-lead ECG** | Highest signal quality, standardized conditions; not portable, needs dedicated space and trained personnel, interferes with occupational duties | **Best for baseline / screening Not recommended for field studies** |
| **Chest strap** | Easy, non-invasive, low-disturbance method, self-applicable, validated at rest and exercise; wireless transmission susceptible to electromagnetic interferences if wireless, only records NN intervals – no real ECG recording | **Most utilized in current field studies Best mix of applicability and validation Recommended** |
| **Wearables (PPG)** | Unobtrusive, high compliance, often with contextual data (activity, sleep, heart rate) for covariate adjustment, proprietary algorithms, motion-prone, raw RR often unavailable, no real HRV recording (PRV) | **Still requires validation Should be seen as exploratory** |

↓

| **3 Software & artifact correction** | |
| --- | --- |
| **Software** must read device raw data  must offer artifact correction  must display RR data for segment evaluation  evaluate features before the project starts  report software name + version  select & report spectral method for frequency-domain parameters | **Artifact correction** threshold-based most common (NN vs. local average)  levels range from very low to very strong (+ custom)  no included study reported the exact threshold level  additional options: noise detection, visual inspection, manual correction  especially important under maritime recording conditions  over-correction → falsely low HRV;  under-correction → falsely high HRV |
| Most frequently used software in the included studies: **Kubios HRV** | |

↓

**4 HRV metrics** – Select metrics that match both the **recording duration** and the **aim of the study -** not every parameter is valid for every recording length

Overview of recommended methodological options for planning heart rate variability (HRV) measurements in field studies, synthesized from the eleven included studies and the guideline literature. Each section combines a general methodological anchor with recommendations derived from the present review. The generic backbone follows established guidance: measurement mode and study concept (1) draw primarily on the Task Force guideline (1996), device choice (2) and its quality criteria on the occupational medicine guideline by Sammito et al. (2024), software and artifact correction are based on general recommendations and considerations and should be adjusted to the specific manual or instructions of the used software (Tarvainen et al. 2021), and HRV metric selection on the same guideline recommendations.

#### References

1. Heart rate variability: standards of measurement, physiological interpretation and clinical use. Task Force of the European Society of Cardiology and the North American Society of Pacing and Electrophysiology. Circulation. 1996;93(5):1043–65.

2. Sammito S, Thielmann B, Klussmann A, Deußen A, Braumann K-M, Böckelmann I. Guideline for the application of heart rate and heart rate variability in occupational medicine and occupational health science. Journal of Occupational Medicine and Toxicology. 2024;19(1).

3. Tarvainen MP, Lipponen J, Niskanen J-P, Ranta-aho PO. Kubios HRV Software User's Guide: Kubios HRV Standard, Kubios HRV Premium (version 3.5). version 3.5 ed. Kuopio: Kubios Oy; 2021.
